# Development and Static Performance Test of EPDM-Encapsulated FBG Sensors for Wind Turbine Blade Deformation Monitoring

**DOI:** 10.3390/mi17060677

**Published:** 2026-05-29

**Authors:** Jianping He, Zhilong Zhou, Tongchun Qin, Qiyu Qu, Haiqin Ding, Hao Wang, Yuping Bao

**Affiliations:** 1 School of Civil Engineering, Nantong Institute of Technology, Nantong 226000, China; 2 School of Information Engineering, Nantong Institute of Technology, Nantong 226000, China; 3 School of Mechanical Engineering, Nantong Institute of Technology, Nantong 226000, China

**Keywords:** ethylene–propylene–diene monomer (EPDM), fiber Bragg grating (FBG), encapsulated sensor, wind turbine blade, static performance, structural health monitoring

## Abstract

Wind turbine blades serve as the core components of wind energy conversion systems, and their safe and stable operation is pivotal to the operational efficiency and reliability of wind farms. However, prolonged operation in harsh environmental conditions such as strong winds, heavy rainfall, ultraviolet radiation, and temperature fluctuations renders wind turbine blades susceptible to fatigue damage and structural failure. Aiming at the drawbacks of traditional electromagnetic sensors, including their vulnerability to lightning strikes and poor corrosion resistance, as well as the elastic modulus mismatch between existing fiber Bragg grating (FBG)-encapsulated sensors and wind turbine blade structures, this study selects the ethylene–propylene–diene monomer (EPDM) as the encapsulation material to develop EPDM-FBG strain sensors. The effectiveness of the proposed sensor in blade strain monitoring is ultimately verified via static load model tests on small-scale wind turbine blades. Test results demonstrate that the EPDM-FBG strain sensor exhibits excellent static strain sensing performance, with its test results being highly consistent with those of bare FBG sensors and a relative error of less than 5%, which can fully meet the practical requirements of static strain monitoring for wind turbine blades. This research provides a novel and reliable monitoring method for the health monitoring of wind turbine blades.

## 1. Introduction

With the continuous advancement of wind power technology and the growing demand for energy conservation and environmental protection, wind power has become an indispensable component of green renewable energy systems. As the core component for capturing wind energy, wind turbine blades are predominantly fabricated from composite materials and operate in harsh outdoor environments for extended periods. Under the combined action of environmental erosion and external loads, blades are prone to the initiation and propagation of defects such as cracks and delamination, which severely jeopardize the safe operation of wind turbines and may even lead to catastrophic accidents. Therefore, it is essential to adopt advanced sensing methods to monitor and detect the mechanical performance of wind turbine blades throughout their service life. Relying on accurate and reliable measurement data, the operational status of wind turbines can be scientifically evaluated, early damage can be detected in a timely manner, maintenance strategies can be dynamically adjusted, and the service performance and service life of wind turbines can be effectively improved.

At present, wind turbine blade monitoring technology has evolved from the traditional “manual inspection” mode to the “sensing monitoring + intelligent analysis” mode, which can be categorized into traditional monitoring technology and novel sensing monitoring technology. Traditional detection technologies mainly include non-destructive testing methods such as manual inspection, robot-assisted inspection, and ultrasonic testing. Manual inspection judges blade damage by visual observation or tapping with the naked eye or binoculars; this method features low cost but suffers from low efficiency, strong subjectivity, and inability to detect hidden internal damages. Unmanned aerial vehicle (UAV) or wall-climbing robot inspection, equipped with high-definition cameras and infrared cameras, can cover blades in remote areas and realize visual detection of surface defects such as cracks and wear. However, this method is greatly affected by severe weather conditions (e.g., heavy rain, strong wind), involves complex image processing procedures, and has an inevitable inspection window period [1,2,3,4]. Ultrasonic testing evaluates internal structural damage by utilizing the reflection characteristics of ultrasonic-guided waves, which can detect millimetric scale defects, but it has poor anti-interference ability, high requirements for operators’ professional skills, and is not suitable for the real-time monitoring of operating blades [5,6].

To realize the real-time online monitoring and performance evaluation of key wind turbine structures, electrical sensing technologies (e.g., resistance strain gauges, piezoelectric ceramics) and optical fiber sensing technology have been widely researched and promoted. Wind turbines, especially offshore ones, operate in extremely harsh marine and outdoor environments. Although electrical sensing technologies offer high testing accuracy and fast response speed, and can monitor multiple parameters such as strain and vibration, they are extremely vulnerable to electromagnetic interference and lightning damage, and have poor corrosion resistance, making them unsuitable for the long-term online monitoring of wind turbine blades [7,8]. In addition to possessing the advantages of conventional electrical sensors, optical fiber sensing technology has prominent merits such as anti-electromagnetic interference, lightning protection, moisture resistance, and the ability to achieve quasi-distributed or fully distributed measurements. At present, it has been successfully applied in civil engineering, aerospace, and other fields such as subgrade, bridge, and aircraft structure monitoring [9,10,11,12,13,14].

In the field of wind turbine blade monitoring, bare optical fibers or fiber Bragg gratings (FBGs) are directly attached to the blade surface for icing monitoring and crack localization [15,16,17,18,19]. Although optical fibers or FBGs, as sensitive elements, exhibit good strain transfer performance when directly arranged on the blade surface, their small diameter and poor shear resistance make them extremely prone to mechanical damage when used directly as sensors. To protect optical fibers or FBGs, metallization packaging and composite material packaging technologies have been developed, and a series of surface-welded and embedded FBG strain sensors have been fabricated, which have been applied in concrete, pavement, and subgrade structures. The accuracy of structural strain testing is highly dependent on the coupling degree between the sensor and the measured structure. Existing FBG sensors with metallization or composite material packaging are usually directly attached to the blade surface by bonding, which easily leads to interface peeling at the bonding position. In addition, metallized FBG sensors are susceptible to corrosion and have poor durability, which limits their application in long-term blade health monitoring.

Rubber materials possess excellent flexibility, elasticity, corrosion resistance, and electrical insulation properties, which can achieve close fitting with the surface of wind turbine blades and effectively transfer the strain generated by blade deformation to FBGs. Therefore, this study selects the ethylene–propylene–diene monomer (EPDM) as the encapsulation material, focuses on the development of EPDM-encapsulated FBG strain sensors, conducts in-depth research on their static sensing performance, and verifies their application effect in the static strain monitoring of wind turbine blades through model tests. This research is expected to provide important technical support for the practical engineering application of FBG sensors in wind turbine blade health monitoring.

## 2. Sensing Principle of Fiber Bragg Grating and Design of EPDM-FBG Strain Sensor

### 2.1. Basic Sensing Principle of Fiber Bragg Grating

A fiber Bragg grating (FBG) is a kind of optical fiber device that forms periodic refractive index changes in the fiber core. When incident light enters the fiber core, only the light with a specific wavelength (called the central wavelength) can be reflected by the FBG, and the rest of the light is transmitted. The central wavelength of the FBG can be expressed by the following formula:

(1) $$\lambda_{B}=2n_{eff}\Lambda$$

where $\lambda_{B}$ is the central wavelength of FBG, $n_{eff}$ is the effective refractive index of the fiber core, and Λ is the grating period.

When the FBG is subjected to external strain or temperature changes, the effective refractive index of the fiber core and the grating period will change, which will lead to a shift in the central wavelength. The relationship between the relative shift in the central wavelength and strain and temperature can be expressed as:

(2) $$\frac{\Delta \lambda_{B}}{\lambda_{B}}=\left(1-\rho_{e}\right)\Delta \varepsilon +\left(\alpha_{f}+\xi_{f}\right)\Delta T$$

where $\rho_{e}$ is the photoelastic coefficient of FBG, Δε is the strain change, $\alpha_{f}$ is the thermal expansion coefficient of the optical fiber, $\xi_{f}$ is the thermo-optic coefficient of the optical fiber, and ΔT is the temperature change.

To further simplify the formula, the strain coefficient $K_{\varepsilon }$ and temperature coefficient $K_{T}$ of the fiber Bragg grating can be introduced, leading to the following expression:

(3) $$\Delta \lambda_{B}=K_{\varepsilon }\Delta \varepsilon +K_{T}\Delta T$$

In practical strain monitoring, temperature compensation is needed to eliminate the influence of temperature on the central wavelength shift. The actual strain compensation method is as follows: an FBG temperature sensor is placed adjacent to the strain sensor. The ambient temperature variation measured by the FBG temperature sensor is substituted into Equation (3), by which the strain value independent of ambient temperature interference can be obtained [20,21].

### 2.2. Selection of EPDM Encapsulation Material

The encapsulation material of the sensor directly determines its sensing performance and service life. For wind turbine blade monitoring applications, the encapsulation material needs to meet the following key requirements: good elasticity, excellent weather resistance, reliable electrical insulation, freeze resistance, wide temperature adaptability, and water resistance. By comparing the comprehensive performance of various common rubber materials (as shown in Table 1), EPDM rubber is selected as the sensor encapsulation material due to its outstanding overall performance. EPDM rubber is a terpolymer synthesized from ethylene, propylene, and non-conjugated diene monomers. It has excellent elasticity (second only to natural rubber) and can maintain good elastic performance at low temperatures. In addition, EPDM rubber exhibits superior weather resistance, ozone resistance, and heat resistance, and can operate continuously at 120 °C, which effectively solves the problem of sensor damage caused by harsh climatic conditions. At the same time, it has excellent electrical insulation performance, which effectively avoids the risk of sensor damage caused by lightning strikes in outdoor environments.

**Figure 1:**
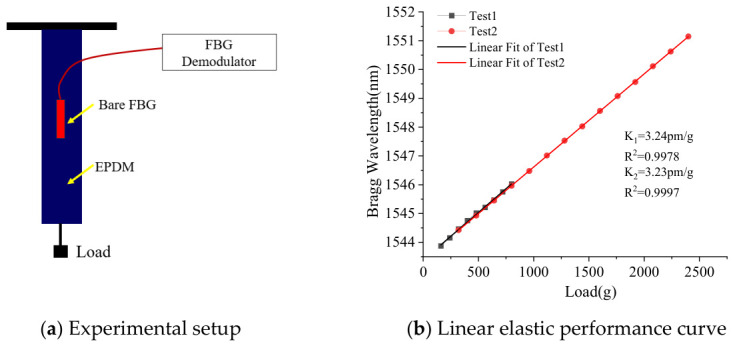
The elastic performance of EPDM.

To verify the elastic performance of EPDM rubber, a uniaxial tensile test was carried out. The EPDM rubber specimen was designed with a length of 11 cm, a width of 1.5 cm, and a thickness of 4 mm. A bare FBG was attached to one side of the specimen; only the two ends of the optical fiber of the bare FBG were bonded by the two-component AB epoxy adhesive. The specimen was suspended vertically, one end was clamped in a universal testing fixture, and weights were hung at the other end to apply a gradual tensile load to the specimen. The load was increased step by step to 2400 g, and the central reflection wavelength of the FBG was recorded after each load was stabilized. Here, the bare FBG with a strain coefficient of 1.2 pm/με is fabricated by ultraviolet (UV) inscription technology and subjected to a tensile strain of 0.8%. Uniaxial tensile tests show that EPDM exhibits stable linear elasticity within the common strain range (<0.8%) for FBG encapsulation (as shown in Figure 1), meeting the requirements for strain transfer. Its upper limit of linear elasticity ranges from 5% to 30%, which is much higher than the working strain range of the sensor, ensuring long-term stable sensing. This result does not emphasize the novelty of the material but verifies the reliability of EPDM as an elastic encapsulation layer for linear strain transfer under small strains, providing a mechanical basis for the design of strain-sensitized structures.

### 2.3. Development of EPDM-FBG Strain Sensor

**Figure 2:**
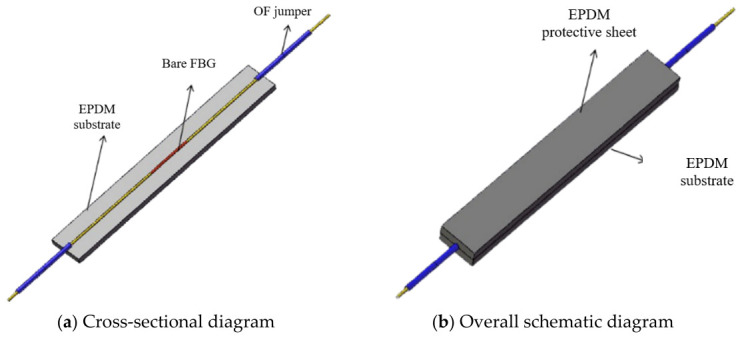
Schematic diagram of EPDM-FBG strain sensor.

The EPDM-FBG strain sensor is mainly composed of an EPDM rubber substrate, an EPDM protective sheet, an FBG sensing element, and an optical fiber jumper. The structural schematic diagram of the sensor is shown in Figure 2. The EPDM rubber substrate is directly bonded to the surface of the wind turbine blade, and its main function is to realize a close fit with the blade and effectively transfer the blade strain to the internal FBG sensing element. The thickness, width, and length of the substrate have a significant impact on the efficiency of the strain transfer sensor. The EPDM protective sheet is used to protect the FBG sensing element from mechanical damage, so its length and width are consistent with those of the rubber substrate, and the thickness is set to 2 mm. The optical fiber sleeve and armored optical cable are used to protect the pigtail of the FBG, and are connected with the optical fiber jumper to form a complete sensing system. Considering that the sensor will be exposed to harsh outdoor environments for a long time, a resin encapsulation shell is designed and fabricated by 3D printing technology to provide secondary protection for the sensor and further improve its environmental adaptability.

**Figure 3:**
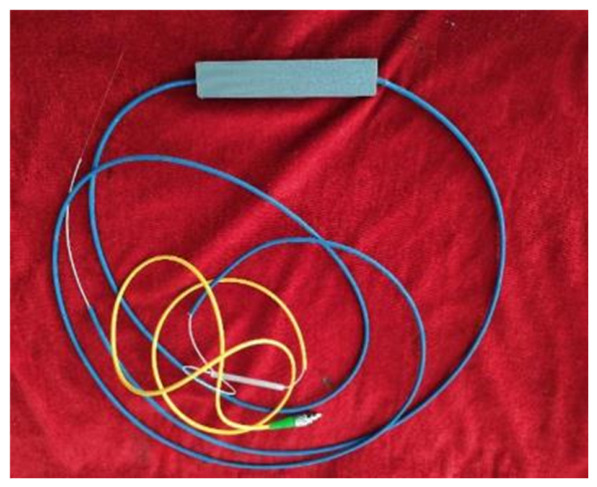
Physical prototype of the EPDM-FBG strain sensor.

Figure 3 shows the physical prototype of the EPDM-FBG strain sensor. The detailed fabrication process of the sensor is as follows: (1) Cut the EPDM rubber into the designed size to prepare the rubber substrate and protective sheet, and press them with a flat steel plate for 24 h to straighten the curved rubber and ensure the flatness of the substrate; (2) open a semicircular groove with an outer diameter of 0.2 mm along the axial direction of the rubber substrate and protective sheet for placing and bonding the FBG, and open a semicircular groove with a radius of 1.5 mm and a depth of 5 mm at both ends of the EPDM sheet for inserting and fixing the optical fiber jumper; (3) clean the impurities and dust from the groove with anhydrous ethanol to ensure the bonding quality, and bond the bare FBG into the axial groove of the rubber substrate, with the grating sensing area accurately located at the geometric center of the substrate; (4) connect the optical fiber jumper to the FBG pigtail, cover the EPDM protective sheet on the substrate, and press it with uniform weight to make the substrate and protective sheet fully bond; (5) bond the 3D-printed resin protective shell on the surface of the EPDM protective sheet to complete the overall encapsulation of the sensor. Here, the EPDM-FBG sensor adopts surface-attached encapsulation, in which the fiber Bragg grating is encapsulated inside the EPDM matrix and the whole sensor is bonded to the surface of the measured structure.

## 3. Static Performance Test of EPDM-FBG Strain Sensor

### 3.1. Strain Sensing Performance Test

**Figure 4:**
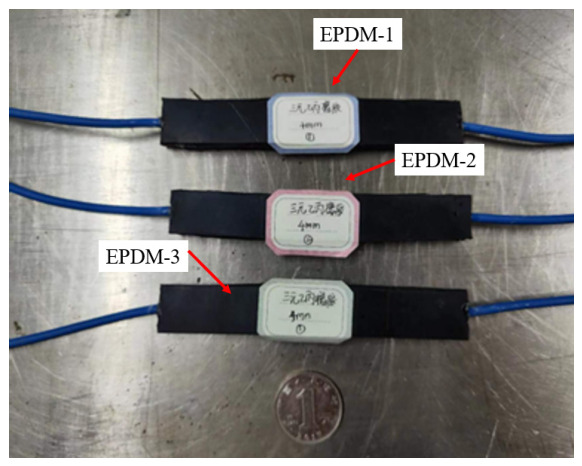
Three EPDM-FBG strain sensors (named as EPDM-1, EPDM-2, EPDM-3).

The strain-sensing characteristics of bare FBG may change due to the influence of the encapsulation material and the fabrication process during the encapsulation process, so it is necessary to calibrate the strain-sensing performance of the fabricated EPDM-FBG strain sensor. Three EPDM-FBG strain sensors (marked as EPDM-1, EPDM-2, and EPDM-3) were fabricated in this study, as shown in Figure 4. The structural parameters of the sensors are as follows: total length 11 cm, EPDM substrate thickness 4 mm, protective sheet thickness 2 mm, and overall width 1.5 cm.

**Figure 5:**
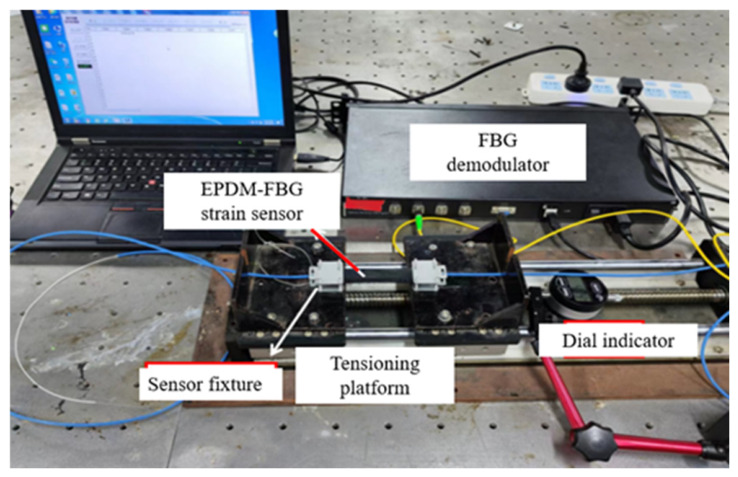
Test setup for the strain-sensing performance of the EPDM-FBG strain sensor.

Figure 5 shows the test setup for the strain sensing performance of the EPDM-FBG strain sensor, which is composed of the EPDM-FBG strain sensor, FBG demodulator, dial indicator, precision tensioning platform, and special sensor fixture. In the test setup, the EPDM-FBG strain sensor is firmly fixed on the precision tensioning platform by using a special sensor fixture, and the effective gauge length of the sensor is set to 8 cm. The FBG demodulator used in the calibration test is the BRX-SFA-C4 model independently developed by Dalian Boruixin Technology Co., Ltd., Dalian, China, which has a test accuracy of ±1.5 pm and a wavelength resolution of 0.01 pm, meeting the high-precision testing requirements of the sensor.

The precision tensioning platform controls the tensile displacement step through the precision turntable on the right side. After each tensile displacement is applied, the actual tensile elongation of the sensor is accurately recorded by the dial indicator with an accuracy of 0.01 mm, and the central reflection wavelength of the sensor is simultaneously recorded by the FBG demodulator. The actual strain of the sensor under each tensile load is calculated by the following formula:

(4) $$\Delta \varepsilon =\frac{\Delta L}{L}$$

where ΔL is the actual elongation of the sensor after each tensile load, and L is the initial effective gauge length of the sensor (8 cm). Each sensor was calibrated three times to ensure the repeatability of the test results, and the strain sensitivity coefficient of the sensor was calculated by drawing the fitting curve of the central wavelength shift and the applied strain.

**Figure 6:**
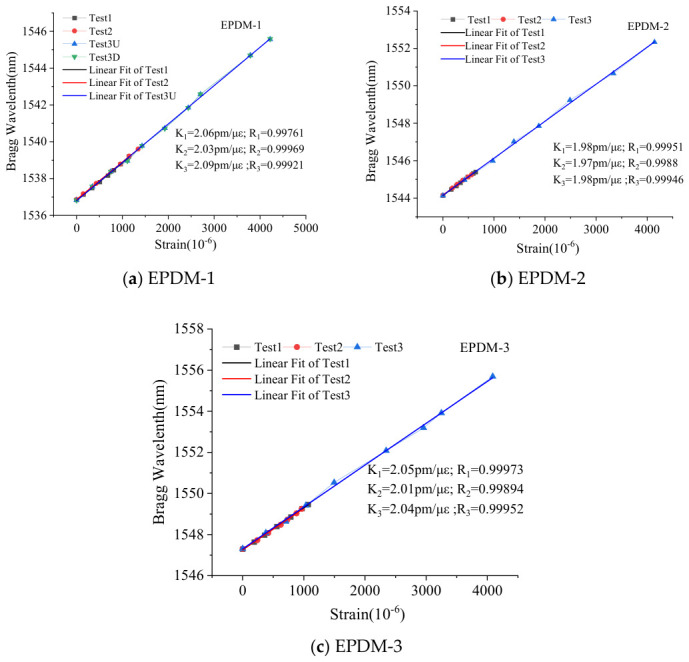
Relationship between the central wavelength shift and the applied strain of EPDM-FBG strain sensors.

Each sensor was subjected to tensile calibration tests repeated three times, and the maximum tensile strain reached more than 4000 με. Figure 6 shows the strain calibration results of the three EPDM-FBG strain sensors. It can be seen from the test results that all three sensors exhibit excellent strain-sensing performance. The strain sensitivity coefficients of EPDM-1, EPDM-2, and EPDM-3 are 2.05 pm/με, 1.98 pm/με, and 2.03 pm/με, respectively, and the linear correlation coefficients of all fitting curves are greater than 0.999, indicating a good linear relationship between the central wavelength shift and the applied strain. Compared with the bare FBG (with a strain sensitivity of about 1.2 pm/με), the strain sensitivity of the EPDM-FBG strain sensor is increased by about 1.7 times, which indicates that the EPDM encapsulation structure has a significant sensitization effect on the FBG strain sensing performance. It can also be seen that when the tensile strain reaches 4500 με, the central wavelength of the FBG basically approaches its ultimate tensile deformation of 0.8%. When the applied strain is loaded up to 5000 με, the encapsulated EPDM still maintains good mechanical performance, whereas the FBG is fractured. Therefore, the ultimate tensile strain of the proposed sensor is approximately 4500 με.

Equation (5) is the formula for the hysteresis error of the sensor:

(5) $$H=\frac{∆\varepsilon_{max}}{\varepsilon_{FS}}\times 100\%$$

Here, H is the hysteresis error; $∆\varepsilon_{max}$ is the maximum difference in output strain between the forward and reverse strokes under the same input load; and $\varepsilon_{FS}$ is the full-scale output strain of the sensor. In Figure 6a, the hysteresis error of the sensor is 0.78%, calculated by using the measured data from both the forward and reverse strokes and Equation (5), which indicates its excellent hysteresis performance. Test3U represents the tensile loading data, and Test3D represents the unloading data.

The strain sensitivity of the EPDM-FBG sensor is approximately 1.7 times higher than that of the bare FBG, which is mainly attributed to two factors: (1) The elastic modulus of EPDM is much lower than that of the optical fiber, so the encapsulation layer produces a strain amplification effect on the FBG under the same structural strain. (2) The encapsulation structure forms a flexible strain transfer interface, enabling the FBG to capture structural deformation more effectively, thus improving the effective strain transfer efficiency. So, this sensitivity improvement is achieved by the combined effect of the encapsulation structure and material matching, rather than the sole contribution of material elasticity.

### 3.2. Temperature Sensing Performance Test

**Figure 7:**
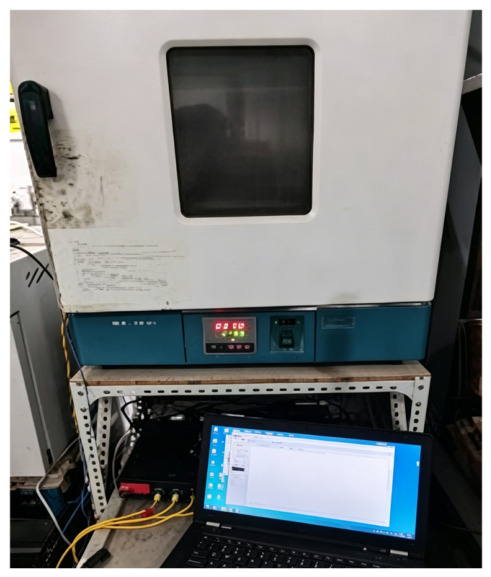
Temperature calibration test setup of EPDM-FBG strain sensor.

Wind turbine blades are usually installed at high altitudes, and the ambient temperature changes greatly during operation. Therefore, the influence of temperature changes on the strain sensing performance must be considered in actual blade strain monitoring. EPDM possesses a higher thermal expansion coefficient than FBG. For FBG encapsulated with EPDM, the overall thermal expansion characteristic is primarily governed by EPDM. In theory, the EPDM-FBG exhibits a greater temperature sensitivity than bare FBG. To investigate the temperature-sensing characteristics of the EPDM-FBG sensor, a temperature calibration test was carried out. The temperature calibration test was carried out by using a constant temperature oven with an accuracy of 0.05 °C, as shown in Figure 7. The EPDM-FBG sensor was placed freely and horizontally inside the temperature chamber, and the FBG demodulator used was the same as that in the strain calibration test to ensure the consistency of the test equipment.

**Figure 8:**
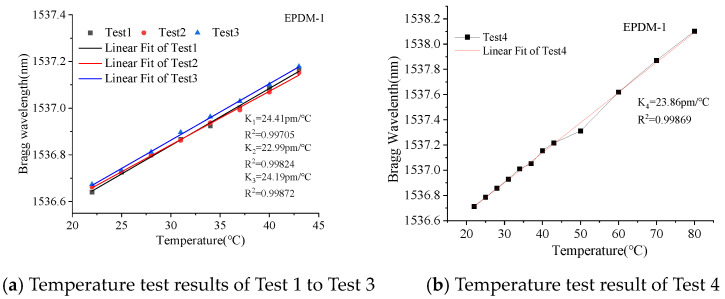
Relationship between central wavelength shift and temperature of EPDM-1 sensor.

Based on the results of the strain calibration test, the three fabricated EPDM-FBG sensors exhibit minimal performance dispersion and good consistency. Therefore, only EPDM-1 was selected for the temperature calibration test. Before the formal test, the oven door was opened to make the internal temperature of the oven consistent with the room temperature (22 °C). The test was carried out at room temperature, the temperature was increased by 3 °C each time, and the central wavelength data of the sensor were recorded after standing for 3 min to ensure the temperature and sensor data were stable. A total of four temperature calibration experiments were carried out. In the first three tests, the temperature was finally increased to 43 °C, and the temperature finally rose to 80 °C in the fourth test. The test results show that the linear correlation coefficient of the EPDM-1 sensor is good, indicating that it has an excellent temperature-sensing performance. The average temperature sensitivity of the sensor is 23.86 pm/℃, which is significantly higher than that of the bare FBG (10.89 pm/℃), as shown in Figure 8.

## 4. Static Load Model Test of Wind Turbine Blade Based on EPDM-FBG Strain Sensor

### 4.1. Test Setup

**Figure 9:**
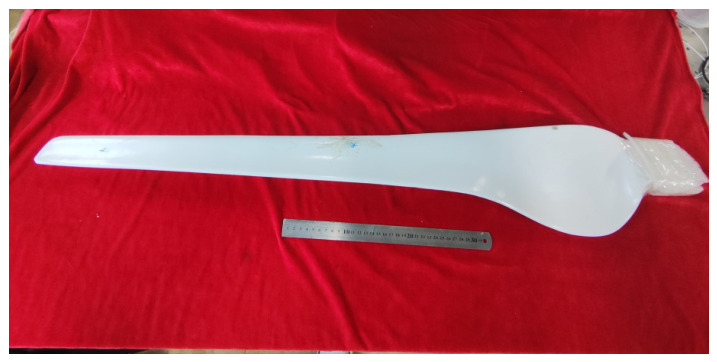
Scaled-down wind turbine blade model for static load test.

Figure 9 shows the scaled-down wind turbine blade model used in the static load test. The total length of the blade model is 96 cm, and the maximum width of the blade surface is 17 cm. The scaled-down blade model is fabricated from fiber-reinforced composite materials, which have the same material mechanical properties as the actual wind turbine blade to ensure the similarity of the test model. The main method of the static load test is to fix the tail end (root) of the blade model, apply a graded static load by hanging weights on the blade surface, and use the EPDM-FBG strain sensors to monitor and analyze the strain changes in the blade model under different loads.

**Figure 10:**
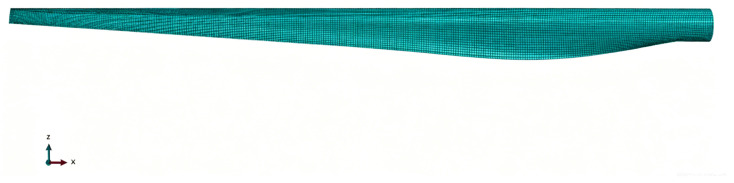
Finite element mesh division of the scaled-down blade model.

**Figure 11:**
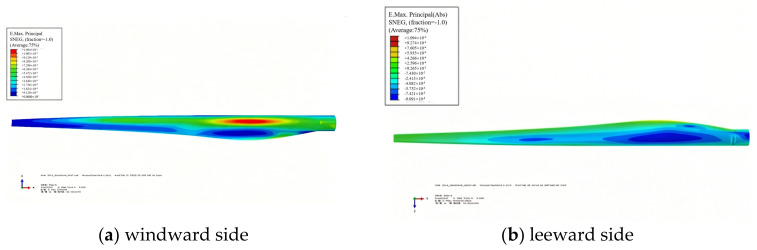
Equivalent strain nephograms of the blade model under a vertical concentrated load.

To determine the optimal placement position of the EPDM-FBG strain sensors and ensure the effectiveness of strain monitoring, finite element analysis (FEA) was carried out on the blade model under static load conditions, as shown in Figure 10. The material parameters of the blade model used in the FEA are as follows: Young’s modulus 45 GPa, shear modulus 18 GPa, Poisson’s ratio 0.25, and density 1950 kg/m^3^, with a grid density of 0.0002 m to ensure the calculation accuracy. After completing the mesh generation of the blade model, to simulate the actual static load test conditions, the blade root was set as the fixed end, and a vertical concentrated load perpendicular to the windward surface of the blade was applied at a position 85 cm from the fixed end. The calculated equivalent strain nephograms of the windward and leeward surfaces of the blade model are shown in Figure 11. Based on the strain nephogram analysis results, the windward surface of the blade is mainly subjected to tensile stress under vertical load, while the leeward surface is mainly subjected to compressive stress. The strain values near the blade root and the middle section are significantly higher than those from the blade trailing edge to the tip, and the strain values of the leading edge are higher than those of the trailing edge under the same load condition.

**Figure 12:**
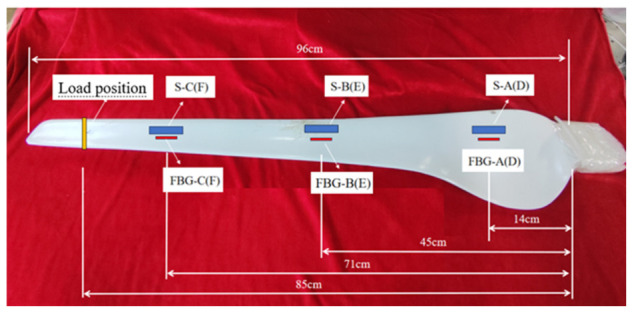
Placement layout of sensors on the windward and leeward surfaces of the blade model.

Based on the finite element analysis results, three EPDM-FBG strain sensors and three bare FBGs (for comparison) were symmetrically arranged on the windward and leeward surfaces of the blade model at the root heights of 14 cm, 45 cm, and 71 cm, as shown in Figure 12. On the windward surface, the three EPDM-FBG strain sensors are named S-A, S-B, and S-C, and the corresponding three bare FBGs are named FBG-A, FBG-B, and FBG-C; on the leeward surface, the three EPDM-FBG strain sensors are named S-D, S-E, and S-F, and the corresponding three bare FBGs are named FBG-D, FBG-E, and FBG-F. This arrangement ensures that the sensors can accurately capture the strain changes in the blade model at key positions under static load. Here, the bare FBG and EPDM-FBG sensors were fixed on the blade surface by full-surface bonding.

### 4.2. Test Results and Analysis

**Figure 13:**
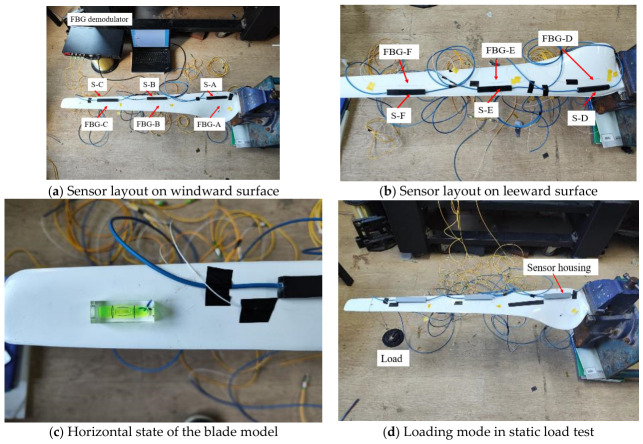
Static loading test apparatus of the wind turbine blade model.

Figure 13 shows the complete static loading test apparatus of the blade model. Each EPDM-FBG strain sensor was firmly bonded to the corresponding marking position on the blade surface, as shown in Figure 12, and the bare FBG sensor was bonded 1 cm below the corresponding EPDM-FBG sensor to ensure that the two sensors were subjected to the same strain under the same load condition. The initial central reflection wavelengths of S-A, S-B, S-C, S-D, S-E, and S-F are 1543.6089 nm, 1535.9219 nm, 1549.0853 nm, 1555.2592 nm, 1537.2175 nm, and 1550.7544 nm, respectively; the initial central reflection wavelengths of FBG-A, FBG-B, FBG-C, FBG-D, FBG-E, and FBG-F are 1536.4832 nm, 1540.2455 nm, 1530.3248 nm, 1536.2761 nm, 1535.7685 nm, and 1529.9499 nm, respectively. The blade model was fixed on a dedicated test rig, and a level was used to adjust the test rig to ensure that the blade model was in a horizontal state before loading, eliminating the influence of its own weight on the test results. At the same time, all deployed sensors were encapsulated with protective shells as shown in Figure 13d, fully simulating the actual engineering application environment of the sensors. The structural parameters of the EPDM-FBG strain sensor used in the test are: length 110 mm, width 15 mm, and substrate thickness 4 mm.

The blade model was fixed at the root, and the static concentrated load was applied at a position 80 cm from the root (consistent with the finite element analysis position). The load was applied in a graded manner, and the load levels were set as 0 g, 163 g, 327 g, 509 g, 678 g, 847 g, 1013 g, 1192 g, and 1349 g. After each load level was applied, the system was left to stand for a certain period of time until the sensor data were completely stable, and then the central reflection wavelength data of each sensor were recorded by the FBG demodulator. In this test, the bare FBG is directly bonded to the blade surface, and the deformation of the blade is directly transmitted to the bare FBG without any intermediate medium, so the strain measured by the bare FBG can be regarded as the true strain of the blade model. In contrast, when the EPDM-FBG strain sensor is bonded to the blade surface, the strain of the blade is first transmitted to the EPDM substrate of the sensor, and then transferred to the internal FBG through the substrate, so there is a certain strain transfer process between the blade and the FBG. As mentioned above, the bare FBG is directly attached to the blade, so the strain measured by the bare FBG can be regarded as the true strain of the blade. It can be seen from the test results that the absolute strain values measured by the bare FBG are larger than those obtained by the EPDM-FBG sensor. The strain transfer efficiency (η) is calculated by the following formula:

(6) $$\eta =\frac{\varepsilon_{EPDM-FBG}^{i}}{\varepsilon_{FBG}^{i}}\times 100\%,i=1,\cdots ,n$$

Here, $\varepsilon_{EPDM-FBG}^{i}$ and $\varepsilon_{FBG}^{i}$ denote the strain measured by the EPDM-FBG sensor and bare FBG under the *i*-th load level.

**Figure 14:**
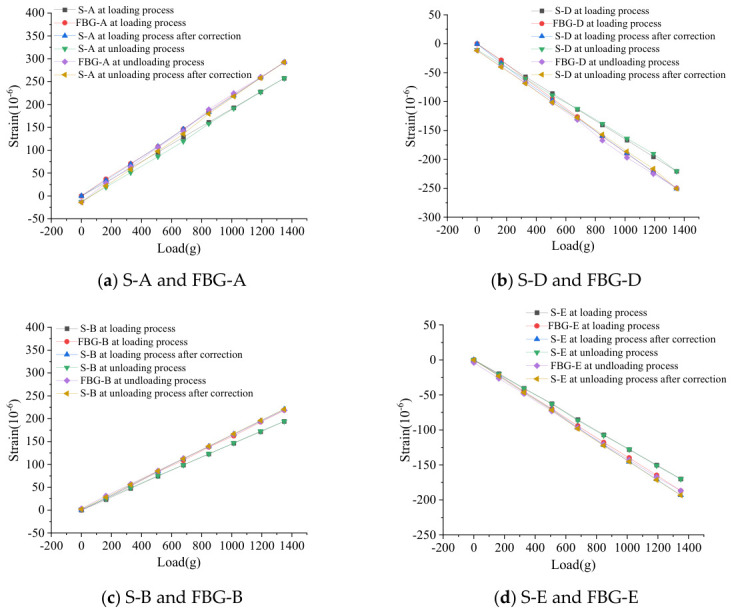

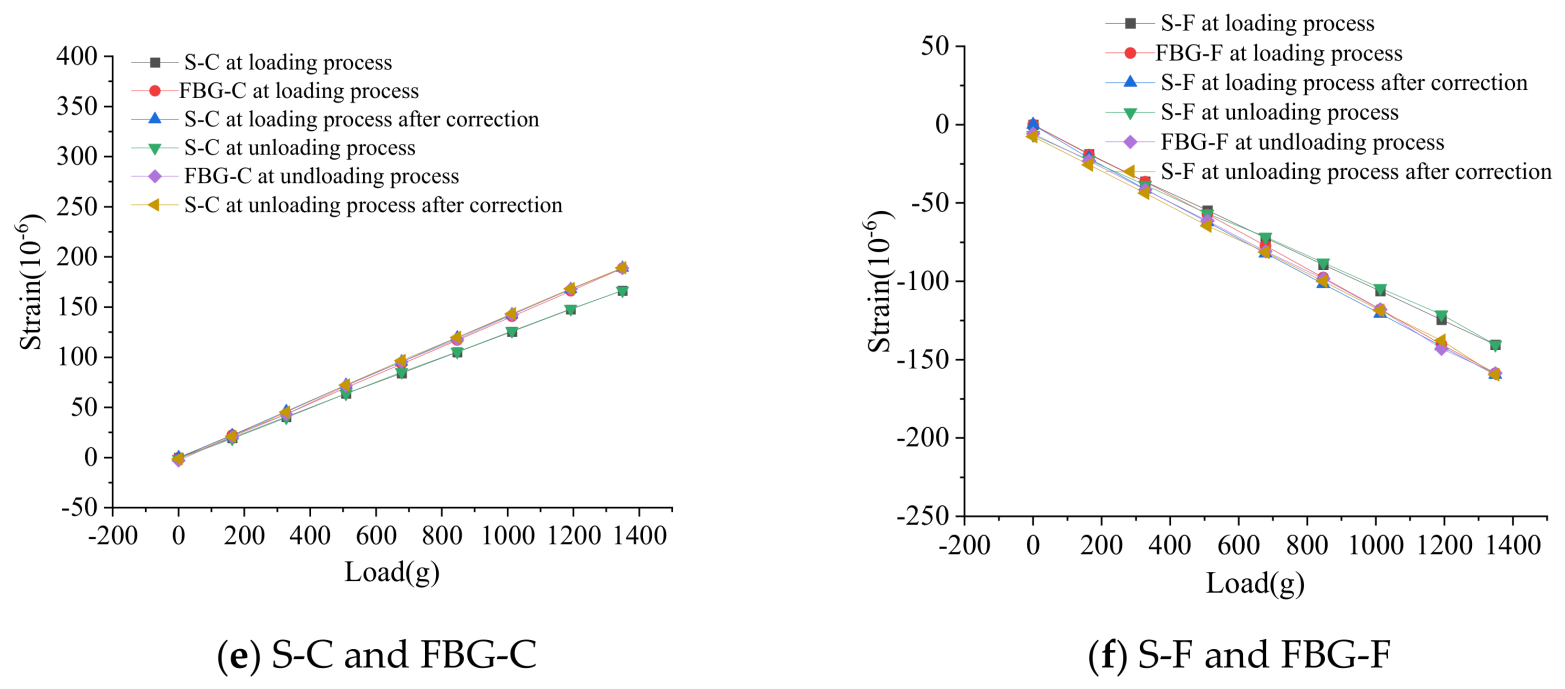
Comparison of the strain measurement results between EPDM-FBG strain sensors and bare FBGs.

**Figure 15:**
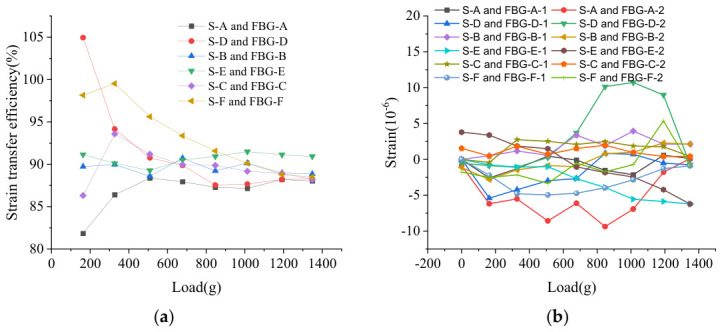
Strain transfer efficiency and error correction curve at different loads. (**a**) Strain transfer efficiency at different loads; (**b**) Absolute errors between the EPDM-FBG and bare FBG.

The bare FBG is directly adhered to the blade surface and acquires true structural strain. As a fully bonded encapsulated sensor, EPDM-FBG suffers strain loss during transmission through the EPDM layer, leading to a lower measured absolute strain value. The 1.7-fold sensitivity promotion means a larger wavelength variation per unit strain, reflecting sensing gain. Lower measured strain magnitude originates from strain transfer attenuation. The two concepts differ physically and have no logical conflict. The sensor is bonded on the whole contact area instead of two ends only, guaranteeing the validity of the strain transfer efficiency calculated by Equation (6). Figure 14 shows the strain measurement results of the EPDM-FBG strain sensors and the corresponding bare FBGs during a complete loading and unloading cycle. Figure 15 illustrates the strain transfer efficiency of the EPDM-FBG sensor under each load level. From the test results as shown in Figure 14 and Figure 15, two conclusions can be obtained: the first one is that in Figure 14, the measured values of the EPDM-FBG sensor exhibit a good linear relationship with the applied load at each load level. This indicates that the EPDM-FBG sensor possesses favorable stability when arranged on the blade. Meanwhile, due to the existence of strain transfer error, the absolute value of the strain measured by the EPDM-FBG sensor is lower than that measured by the bare FBG; the second one is that the strain transfer efficiency ranges from 87% to 89%, excluding the first load level, and the average value is determined as 88%, as shown in Figure 15. After the correction of strain transfer error, the strain measured by the EPDM-FBG sensor is basically consistent with that of the bare FBG, with the absolute error less than 10με as shown in Figure 15b. In Figure 15a, “S-A and FBG-A” in the figure indicates that the corresponding strain transfer efficiency curve is calculated from the sensor S-A and FBG-A. In Figure 15b, “S-A and FBG-A-1” denotes the absolute errors between the strain measured by EPDM-FBG after correction and that measured by the bare FBG during the loading process, and “S-A and FBG-A-2” denotes that at the unloading process. The other labels follow the same definition.

**Figure 16:**
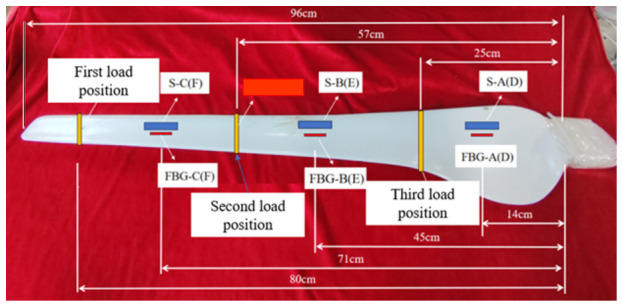
Schematic diagram of the multi-point loading positions on the blade model.

During actual operation, wind turbine blades are often subjected to various adverse loads such as foreign object impact, uneven icing, and lightning strikes. Timely identification of the location of such adverse loads or structural damage on the blades can significantly improve the maintenance efficiency of wind turbines and mitigate potential safety risks. To verify the ability of the EPDM-FBG strain sensor to identify the load position of the blade, multi-point loading tests were designed to capture the strain distribution curves of the blade model at different loading positions through the arranged sensors. The test procedure is as follows: a fixed load of 1200 g was applied at the positions 80 cm, 57 cm, and 25 cm from the blade root, respectively. The central reflection wavelength of each sensor was recorded at each load position, and then the corresponding strain values were calculated by using the pre-calibrated strain sensitivity coefficients of the sensors, as shown in Figure 16. The loading sequence was arranged as follows: apply the load at the first loading point, hold it for a certain period and then unload; after the blade model returns to the free state, apply the load at the second loading point, maintain the same holding time and then unload; once the blade model fully recovers to the free state, apply the load at the third loading point.

**Figure 17:**
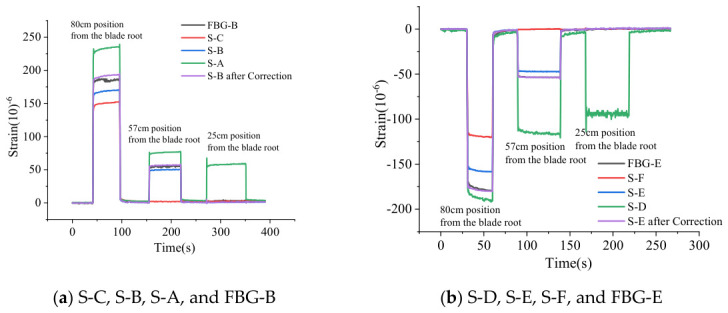
Strain measurement results of the blade model under multi-point loading conditions.

The strain changes in the blade model measured by each sensor were calculated based on the central wavelength values recorded during the entire test process, and the strain change curves of each sensor under multi-point loading conditions were plotted, as shown in Figure 17. Based on the strain monitoring data of the windward surface sensors, the following conclusions can be drawn: under the first loading condition (loading point near the blade tip), the strain values measured by S-A, S-B and S-C all show significant changes, indicating that the load near the blade tip causes overall deformation of the blade model; under the second loading condition (loading position between S-B and S-C), S-B and S-A measure significant strain changes, while the strain value measured by S-C is basically zero, which is consistent with the mechanical deformation characteristics of the cantilever beam structure; under the third loading condition (loading point near the blade root, i.e., the fixed end), the deformation of the blade tip is minimal due to the constraint of the fixed end, and only S-A can measure significant strain changes. In the monitoring data, it can be seen that the measured strains of the EPDM-FBG sensors (S-E and S-B) agree well with those obtained by the bare FBGs (FBG-E and FBG-B), which further verifies that the proposed sensor is well applicable to the deformation monitoring of wind turbine blades. In this verification test, the demodulation device is limited to only four channels, making it impossible to connect all sensors simultaneously. To ensure the continuity of the experiment, the monitoring data of the bare FBG-C, FBG-F, FBG-A, and FBG-D were not acquired in this test; only the data of EPDM-FBG sensors, FBG-B, and FBG-E were retained.

## 5. Conclusions

Aiming at the technical problems of traditional electromagnetic sensors and existing FBG-encapsulated sensors in wind turbine blade health monitoring, this study independently develops an EPDM-encapsulated FBG strain sensor and conducts in-depth and systematic research on its structural design, static sensing performance, and practical application effect through model tests. The main research conclusions are as follows:

(1) Based on the performance requirements of wind turbine blade monitoring, EPDM rubber is selected as the sensor encapsulation material due to its excellent comprehensive performance (including good elasticity and reliable electrical insulation). A reasonably structured EPDM-FBG strain sensor is designed and fabricated, and a 3D-printed resin protective shell is adopted for secondary protection of the sensor, which significantly improves the environmental adaptability and durability of the sensor in harsh outdoor conditions.
(2) Static performance test results show that the EPDM-FBG strain sensor has excellent strain and temperature sensing performance. The average strain sensitivity of the sensor is 2.02 pm/με, which is 1.7 times that of the bare FBG, indicating a significant sensitization effect; the average temperature sensitivity is 23.86 pm/℃, and the linear correlation coefficients of both strain and temperature sensing are greater than 0.998, showing good linear sensing characteristics.
(3) Static load model tests on the small-scale wind turbine blade show that the EPDM-FBG strain sensor can accurately monitor the static strain of the blade model, and its test results are highly consistent with those of the bare FBG sensor, with a relative error of less than 5%. The sensor also exhibits excellent repeatability and stability under graded loading conditions, which can fully meet the actual engineering requirements of static strain monitoring for wind turbine blades. In addition, multi-point loading tests verify that the sensor can effectively capture the strain distribution characteristics of the blade under different load positions, which provides a technical basis for subsequent blade-load position identification and damage localization.

The EPDM-FBG strain sensor developed in this study has the advantages of a simple structure, low fabrication cost, good environmental durability, and high accuracy, which provides a novel and reliable technical method for the static health monitoring of wind turbine blades and has important engineering application value and promotion prospects. In subsequent research, we will conduct further in-depth studies on the thickness optimization of the sensor encapsulation layer, establish a refined strain transfer model of the EPDM-FBG sensor to further optimize the sensor structural dimensions and improve the strain transfer efficiency. At the same time, we will carry out dynamic vibration tests on wind turbine blades, explore the dynamic sensing performance of the sensor under vibration conditions, and study the damage identification method of wind turbine blades under dynamic load conditions to further expand the engineering application scope of the sensor.

## Figures and Tables

**Table 1:** Comparison of properties of different kinds of rubber.

Rubber Type	Elasticity	Weather Resistance	Insulation	Freeze Resistance	Temperature Resistance	Water Resistance
Natural Rubber (NR)	Excellent	Medium	Good	Good	Good	Medium
Butyl Rubber (IIR)	Medium	Good	Good	Good	Good	Good
Ethylene–Propylene–Diene Monomer (EPDM)	Good	Good	Good	Good	Good	Excellent
Nitrile Rubber (NBR)	Poor	Medium	Poor	Poor	Good	Good

## Data Availability

The data presented in this study are available on request from the corresponding author.
